# The Effect of Heat Treatment on the Microstructure and Mechanical Properties of 2D Nanostructured Au/NiFe System

**DOI:** 10.3390/nano10061077

**Published:** 2020-05-31

**Authors:** Tatiana Zubar, Valery Fedosyuk, Daria Tishkevich, Oleg Kanafyev, Ksenia Astapovich, Artem Kozlovskiy, Maxim Zdorovets, Denis Vinnik, Svetlana Gudkova, Egor Kaniukov, Antonio Sergio B. Sombra, Di Zhou, Rajshree B. Jotania, Charanjeet Singh, Sergei Trukhanov, Alex Trukhanov

**Affiliations:** 1 Laboratory of Magnetic Films Physics, Scientific-Practical Materials Research Centre of National Academy of Sciences of Belarus, 220072 Minsk, Belarus; fix.tatyana@gmail.com (T.Z.); fedosyuk@physics.by (V.F.); dashachushkova@gmail.com (D.T.); olegkan96@mail.ru (O.K.); ks.astapovich@gmail.com (K.A.); truhanov86@mail.ru (A.T.); 2 Laboratory of Single Crystal Growth, South Ural State University, 454080 Chelyabinsk, Russia; denisvinnik@gmail.com (D.V.); svetlanagudkova@yandex.ru (S.G.); ka.egor@mail.ru (E.K.); 3 The Institute of Nuclear Physics, Almaty 050032, Kazakhstan; artem88sddt@mail.ru (A.K.); mzdorovets@gmail.com (M.Z.); 4 Engineering Profile Laboratory, L.N. Gumilyov Eurasian National University, Nur-Sultan 010008, Kazakhstan; 5 Department of Intelligent Information Technologies, Ural Federal University named after the First President of Russia B.N. Yeltsin, 620075 Yekaterinburg, Russia; 6 SEC “Nanotechnology”, Moscow Institute of Physics and Technology (State University), 141701 Dolgoprudny, Russia; 7 Department of Physics, Federal University of Ceara, 60-455-970 Fortaleza, Brazil; asbsombra@gmail.com; 8 Electronic Materials Research Laboratory, Key Laboratory of the Ministry of Education & International Center for Dielectric Research, School of Electronic Science and Engineering, Xi’an Jiaotong University, Xi’an 710049, China; zhoudi1220@gmail.com; 9 Department of Physics, Electronics and Space Science, Gujarat University, Ahmedabad 380009, India; rbjotania@gmail.com; 10 School of Electronics and Electrical Engineering, Lovely Professional University, Phagwara 144411, India; rcharanjeet@gmail.com; 11 Department of Technology of Electronics Materials, National University of Science and Technology MISiS, Leninsky Prospekt, 4, 119049 Moscow, Russia

**Keywords:** pulsed electrodeposition, multilayer system, NiFe nanograins, heat treatment, surface microstructure, nanohardness, Young’s modulus, elastoplastic deformation

## Abstract

Nanostructured NiFe film was obtained on silicon with a thin gold sublayer via pulsed electrodeposition and annealed at a temperature from 100 to 400 °C in order to study the effect of heat treatment on the surface microstructure and mechanical properties. High-resolution atomic force microscopy made it possible to trace stepwise evolving microstructure under the influence of heat treatment. It was found that NiFe film grains undergo coalescence twice—at ~100 and ~300 °C—in the process of a gradual increase in grain size. The mechanical properties of the Au/NiFe nanostructured system have been investigated by nanoindentation at two various indentation depths, 10 and 50 nm. The results showed the opposite effect of heat treatment on the mechanical properties in the near-surface layer and in the material volume. Surface homogenization in combination with oxidation activation leads to abnormal strengthening and hardening-up of the near-surface layer. At the same time, a nonlinear decrease in hardness and Young’s modulus with increasing temperature of heat treatment characterizes the internal volume of nanostructured NiFe. An explanation of this phenomenon was found in the complex effect of changing the ratio of grain volume/grain boundaries and increasing the concentration of thermally activated diffuse gold atoms from the sublayer to the NiFe film.

## 1. Introduction

Soft magnetic NiFe films are used in a wide range of applications due to the optimal balance of electric, magnetic and mechanical properties [1,2,3]. Permalloy has applications in areas, such as low frequency magnetic shielding and transformer cores, due to the successful combination of functional properties (high permeability, low coercivity, and small magnetic anisotropy) [4,5,6,7,8]. In addition, permalloy films are widely used as functional magnetic materials in magnetic field sensors (since they demonstrate the effects of giant and amorphous magnetoresistance) [9,10,11], magnetic recording devices [12,13], as spintronic material and as electromagnetic shields to protect functional electronics from permanent magnetic fields and electromagnetic radiations [14,15]. Ni-Fe alloys also show good corrosion resistance [16], good adhesion to various types of the substrates [7,14,15], and excellent mechanical characteristics (hardness in macro- and nanoscale, elastic modulus, wear and crack resistance, as well as resistance to plastic deformation) [17,18]. Thanks to this, permalloys or Ni-rich alloys are an attractive material for use as a functional coating that provides both mechanical protection and protection from the electromagnetic and magnetic fields. Electrodeposited films are promising, due to high economic viability of the electrodeposition process. The ability to deposit thin films and coatings via electrodeposition on puff-shaped substrates should also be noted among the main advantages of the method. This is especially important for using magnetic films as shields from electromagnetic interference, when it is necessary to cover parts. Until now, internal stresses are the unresolved problem of deposited NiFe films and coatings. Internal stresses are greater, the thicker the film and the more complex the shape of the substrate. Residual stresses in the film and coating can lead to a decrease in the magnetic and electrical properties and, of course, mechanical characteristics. 

There are many ways to reduce stresses in electrodeposited alloys. In addition, an increase in current density or iron content leads to an increase in residual stress in the NiFe system [19]. Electrolyte additives have been specially developed that are able to influence the structure of the alloy during synthesis [20,21,22]. Varying the concentration of main components is also used to reduce the internal components. For example, the NiCl component is typically used to increase the mobility of metal ions during the deposition of the NiFe alloy and, at the same time, to increase the film growth rate. The authors of the article [23,24] proved that an excessive concentration of chlorine ions leads to the formation of an alloy with a low coercive force and high internal stresses. Heat treatment of the NiFe alloy and coatings also remains a promising way to reduce internal stresses, which is widely shown in publications [25,26,27,28,29]. In addition, heat treatment in air activates surface oxidation. As a rule, the natural oxide layer does not affect the mechanical properties [30,31,32]. Although specially created oxide can not only harden, but even perform the function of a wear-resistant layer with enhanced mechanical characteristics [33,34]. Despite this, there is no work that would study the effect of the oxide layer of a nanostructured NiFe film on its mechanical properties. Moreover, thorough studies of microstructural characteristics and mechanical properties of the near-surface layer after intentional oxidation have not been carried out.

In this article, we studied the effect of heat treatment on the microstructure and mechanical properties of nanostructured NiFe films that are deposited on a Si wafer with an Au sublayer. We used the temperature range of heat treatment from 100 to 400 °C, since, based on studies [34,35,36,37,38], critical changes in the mechanical and magnetic properties occur in this range. Heat treatment at higher temperatures leads to changes in the crystal lattice [25,26,27,29], for example, polymorphic transformations. We have shown that even relatively low temperatures can have a beneficial effect on the hardness, Young’s modulus, and resistance to the elastoplastic deformation. In addition, we studied differences in the behavior of the surface layer and the internal volume of the material and found explanations for their opposite response to an increase in heat treatment temperature.

## 2. Materials and Methods 

A single-crystal silicon wafer with a crystallographic orientation (100) was used as a substrate for the formation of the Au/NiFe system. A 100 nm thick gold film was obtained by magnetron deposition in order to provide sufficient conductivity for stable electrodeposition of a NiFe film. Based on our previous work [36,37], it is known that such a gold sublayer has a quasi-amorphous structure with a roughness of less than 1 nm. This gold sublayer is excellent for electrolytic deposition of magnetic films, since it has high conductivity and NiFe films do not inherit the structure of gold. 

Pulsed electrodeposition was used to form a nanostructured NiFe film. A short pulse duration (10^−3^ s) leads to the formation of a material with a small grain size. This is due to the fact that grains nucleate and grow during the pulse time (10^−3^ s) and the growth process stops after the end of the current supply. New crystallization centers appear when the current is turned on the next time (second pulse). Thus, the choice of pulse duration allows for you to select the desired grain size. More detailed control of the microstructure by varying the pulse duration is described in the previous article [38,39,40]. Complex electrolyte was used for NiFe electrodeposition. The composition of electrolyte and main parameters, such as pH level, electrolyte temperature, current density, and durations of the pulse and pause, are given in Table 1.

Heat treatment was carried out at temperatures of 100, 200, 300, and 400 °C in air. The samples were heated at a rate of 100 °C/h, and then kept at maximum temperature for 1 h and naturally cooled to room temperature. 

Table 2 provides the main features of the as-prepared Au/NiFe nanostructured system (without heat treatment). The total thickness of the system is about 700 nm (100 nm of the Au film plus 600 nm of the NiFe film). The chemical composition of the ratio between nickel and iron was investigated while using the Energy-dispersive X-ray spectroscopy (EDX) by Rigaku Inc. spectrometer (Tokyo, Japan) in the previous work [41]. Thus, a nickel- iron alloy obtained under the conditions described above contains approximately 24 at.% of iron and 76 at.% of nickel. The crystal structure was also investigated in detail in [41] at room temperature while using the DRON-3M X-ray diffractometer (XRD) (Burevestniuic Inc., St. Petersburg, Russia) with Co-Ka (*λ* = 0.178 nm). It was found that as-prepared NiFe film is single-phase isostructural samples, which are well described by a cubic face-centered lattice and the space group Fm3m (No. 225).

The surface microstructure of the NiFe film was investigated while using atomic force microcopy (AFM) mode of the Hysitron 750 Ubi device (Hysitron Inc., Eden Prairie, MN, USA). The grain size distribution was plotted using statistical analysis of the AFM images. At least five images with size 3 × 3 and 10 × 10 μm^2^ were used. The following equation calculated the proportion of grain area:

(1) $$P_{i}=0.25\frac{d_{i}^{2}\pi n_{i}}{S}$$

where *d_i_* is the equivalent disk diameter of a given grain, *S* is the total area of all grains in the analyzed image, and *n_i_* is the number of grains with a given size. The most probable grain size was determined as the values corresponding to the maxima of the Gaussian functions (Gauss fit) describing the size distribution plots. 

The measurement of the micromechanical parameters was studied using the nanoindenter Hysitron 750 Ubi by penetration of the Berkovich diamond pyramid with continuous registration of the deformation curves (indentation load (*P*) vs. indentation depth (*h*)). The high-precision nanoindentation method allows for us to study the response of nanomaterials to mechanical deformation. Not only elastic modulus and hardness can be estimated, but also stiffness, resistance to mechanical deformation, wear resistance, films adhesion, as well as the effect of crack toughness, phase transitions, polymorphic transformations, and much more on the mechanical properties [42,43,44]. Therefore, nanoindentation was chosen by us as the main method for studying the mechanical properties of nanostructured permalloy. At least 25 measurements were carried out for each value of the maximal indentation depth and for each sample. Each deformation curve included 2–4 thousand measurements. The maximal values of the indentation depth were 10 nm (for the near-surface layer investigation) and 50 nm (for the investigation of the material in bulk). The penetration of the indenter to a depth of more than 10% of the film thickness might not correctly reflect the mechanical properties, since the influence of the substrate increases during increasing the indentation depth [45,46]. The materials were loaded according to the scheme “10-10” (10 s of loading, 10 s of unloading). Calibration of the nanoindenter was done immediately before the experiment. A standard sample of polished fused quartz with known constant values of hardness and Young’s modulus was used for calibration. Calibration was carried out in the range of indentation depths from 2 to 100 nm. The calculation of the micromechanical properties was carried out using the Oliver–Farr method [47]. 

The inter-diffusion process of gold atoms into a NiFe film was calculated according to the basic diffusion equation (the I Fick’s law [48]) and the Arrhenius equation [49,50]. Equation (2) determined the temperature dependence of the diffusion coefficient (*D*):

(2) $$D=D_{0}\exp\left[-\frac{Q}{T}\right]$$

where *D*_0_—the diffusion factor, *Q*—the activation temperature of the diffusion process corresponding to the activation energy, and *T*—thermodynamic temperature. For gold atoms diffusing in the NiFe film, *D*_0_ = 1.01 × 10^−3^ cm^2^/s, *Q* = 17,750 K, *T* = 293–673 K. The relative concentration of gold atoms in the NiFe layer (*C_Au_(x,t)*) at a distance x from the Au/NiFe interface to time *t* was calculated while using Equation (3) based on the I Fick’s law:

(3) $$C_{Au}\left(x,t\right)=\frac{C_{0}}{\sqrt{\pi Dt}}exp\left[-\frac{x^{2}}{4Dt}\right]$$

where *C*_0_ is the concentration of gold atoms at the Au/NiFe interface, *D* is the temperature diffusion coefficient of gold atoms diffusing in the NiFe layer, *D*_0_ is the diffusion factors. In present work, and *C*_0_ = 1.

## 3. Results and Discussion 

**Figure 1:**
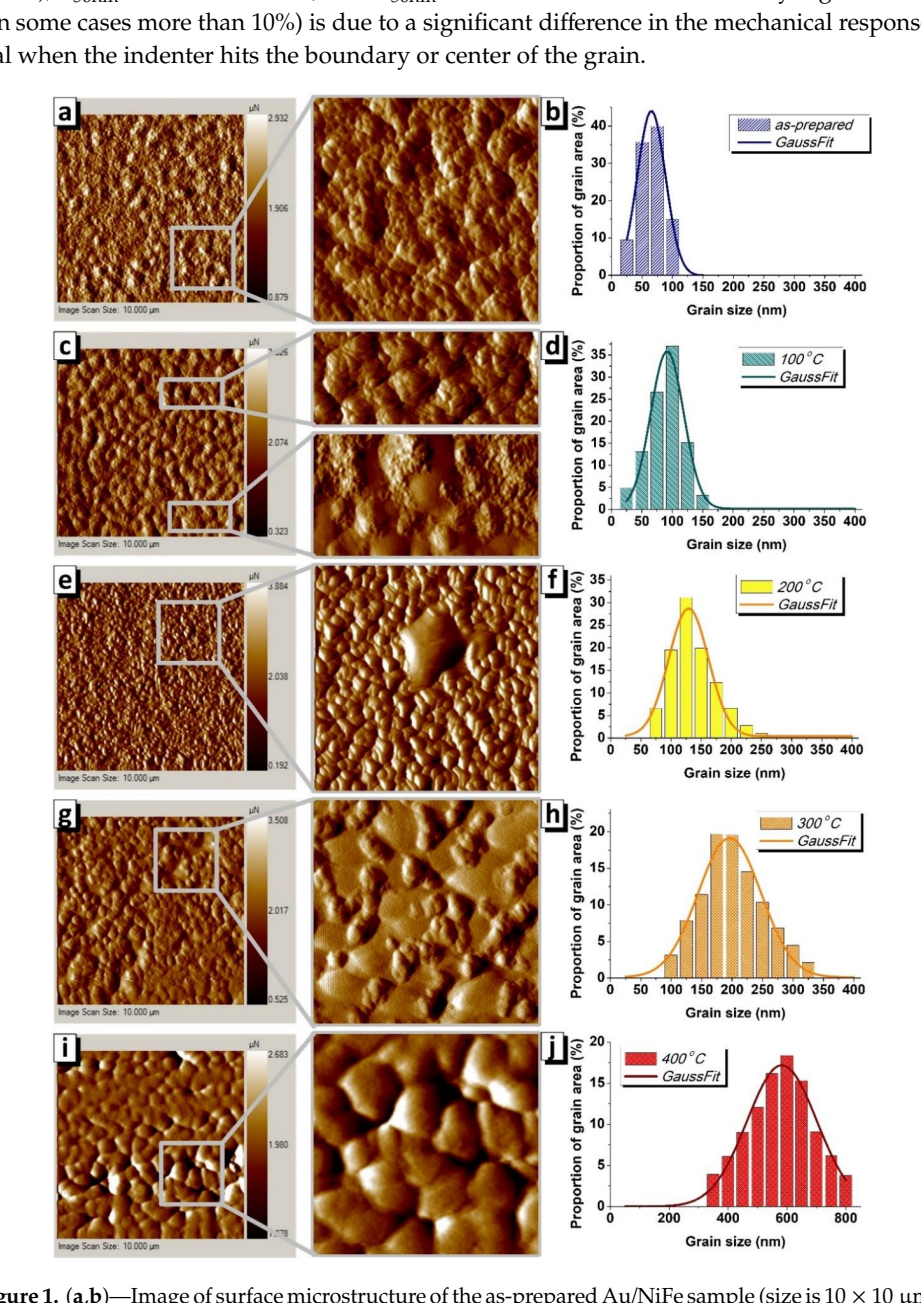
(**a**,**b**)—Image of surface microstructure of the as-prepared Au/NiFe sample (size is 10 × 10 µm^2^) and enlarged fragment (size is 3 × 3 µm^2^) obtained using AFM mode with grain size distribution. (**c**,**d**)—the same for the Au/NiFe sample after heat treatment at *T* = 100 °C. (**e**,**f**)—the same at *T* = 200 °C. (**g**,**h**)—the same at *T* = 300 °C, (**i**,**j**)—the same at *T* = 400 °C. Images were obtained immediately after synthesis or heat treatment without oxide removal.

Figure 1 shows microstructure evolution of the surface of NiFe films under the influence of heat treatment. The surface of the as-prepared sample looks uniform (Figure 1a). The sizes of all grains are close to each other and they are in a narrow range from 30 to 100 nm (Figure 1b). It is noticeable in the full AFM image and the enlarged fragment that small grains tend to combine and form clusters with a size of 1–2 µm.

**Figure 2:**
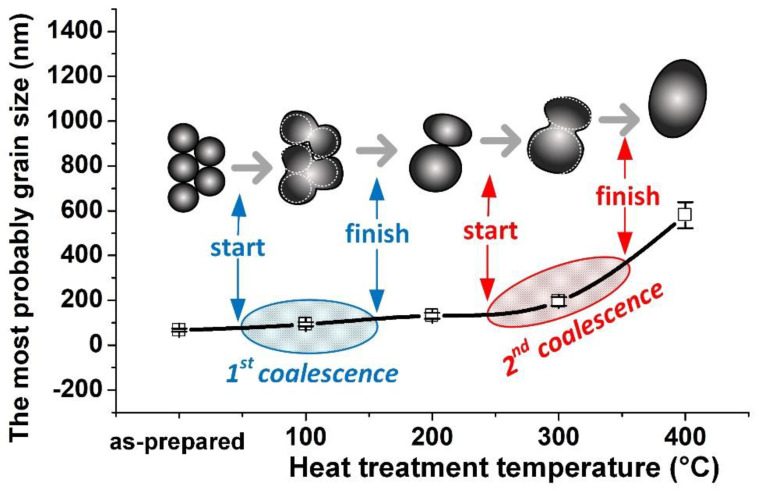
An increase in the most probable grain size upon heat treatment during two stages of coalescence. The most probable grain size was defined as the maximum of the function describing the grain size distribution in accordance with the Gauss law.

The cluster structure becomes more pronounced after heating at 100 °C. Each cluster with a size of 1–2 μm contains several tens of grains, which is well visualized in the AFM image and an upper enlarged fragment (Figure 1c). In addition to coalescence of grains, their enlargement is observed. Figure 1d shows the grain size distribution of the NiFe film after heat treatment at 100 °C. Figure 2 shows the dependence of the most probable grain size—which corresponds to the maximum of the Gauss function—on the heat treatment temperature. The results showed that heat treatment at 100 °C leads to an increase in size from 58 to 93 nm. In addition, there is center of gomogemization (see, lower enlarged AFM fragment). 

The coalescence process is completed when the heat treatment temperature reaches 200 °C. Figure 1e shows distinct individual grains that are not clustered. The most probable grain size is 13 nm. The dispersion width increases with an increasing grain size, as can be seen in Figure 1f. With a further increase in temperature to 300 °C, the second stage of coalescence begins, which will be completed between 300 and 400 °C. The formation of multigrain clusters is also characteristic of the second stage of coalescence, as well as of the first. The presence of a large number of smooth sections around the clusters suggests that the second stage of grain association is close to completion. Smooth areas with a homogeneous surface are likely to become grains, the boundaries of which will be at the place of clusters. The grain size approaches 200 nm for the sample that was subjected to heat treatment at 300 °C (we mean grains inside the clusters, since it is difficult to determine the boundaries of large smooth grains). An increase in the temperature to 400 °C leads to a significant increase in the most probable size of grains to 580 nm. Obviously, the coalescence is complete and the surface looks uniform in this sample. The evolution of the grain microstructure, including two stage of the grain coalescence, are presented schematically in Figure 2.

**Figure 3:**
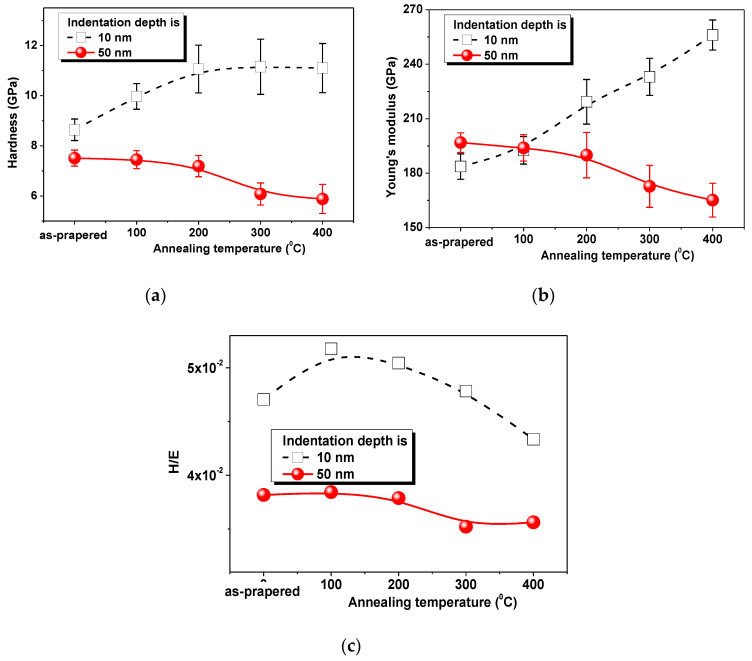
Mechanical properties of the Au/NiFe nanostructured system with indentation depth of 10 nm (black squares) and 50 nm (red circles) after heat treatment investigated using nanoindentation. (**a**) Dependence of the hardness, (**b**) Young’s modulus, and (**c**) resistance to the elastoplastic deformation on the heat treatment temperature.

Figure 3 shows the results of the nanoindentation test. There are two main parameters that characterize the mechanical properties, hardness (*H*) and Young’s modulus (*E*). Improving mechanical properties always remains an urgent task of materials science. However, it is important not only to increase *H* and *E*, but to maintain their balance. The ratio of *H*/*E* (Figure 3c) characterizes the stability of the material under elastoplastic deformation. The ideal case for thin films and coatings is when the ratio *H*/*E* is 0.1. Low hardness with an excessively high modulus softens the material, and the opposite case leads to brittleness [51,52,53,54,55].

The mechanical tests were carried out at two depths—10 nm (black squares) and 50 nm (red circles). Thus, we tried to compare the behavior of the surface and volume of a NiFe film under the influence of heat treatment. The results of nanoindentation show that heat treatment has an opposite effect on the surface layer and the internal volume of the material. Analysis of graphs in Figure 3a,b allows for us to conclude that the hardness and Young’s modulus of the as-prepared sample on the surface and in the bulk are quite close *H*_10nm_ = 8.64 ± 0.43 GPa, *H*_50nm_ = 7.51 ± 0.32 GPa and *E*_10nm_ = 183.6 ± 7.0 GPa, *E*_50nm_ = 196.8 ± 5.4 GPa. An increase in heat treatment temperature leads to a nonlinear increase in Young’s modulus to 256.1 ± 8.3 GPa (for 400 °C) and hardness to 11.1 ± 0.98 GPa on the surface (black squares) and a simultaneous decrease in these parameters in the film volume (red circles), *E*_50nm_ = 165.1 ± 9.3 GPa, and *H*_50nm_ = 5.88 ± 0.58 GPa. The relatively high measurement error (in some cases more than 10%) is due to a significant difference in the mechanical response of the material when the indenter hits the boundary or center of the grain.

The ratio *H*/*E* (Figure 3c) or resistance to the elastoplastic deformation during elastoplastic deformation inside the material (*h* = 50 nm) tends to nonlinearly decrease. The ratio remains almost unchanged during heat treatment not higher than 200 °C (*H*/*E* = 0.037–0.038), and then decreases to 0.035. The surface layer during heat treatment at 100 °C has an increase in deformation resistance from 0.047 to 0.051, and a decrease to 0.043 with an increase in temperature to 400 °C.

Probably, the opposite behavior of the mechanical properties is associated with the difference in the processes activated by heat treatment, both in the material volume and on the surface. We concluded that three complex processes significantly affect the mechanical properties of the system after analyzing the investigation results and literature [24,25,26,27,30,35,39,48]:

1. An increase in grain size, which is always accompanied by a decrease in the number of grain boundaries.

2. The formation of oxide on the surface.

3. Thermally activated diffusion of gold atoms from the sublayer into the film.

The authors did not come to a consensus on the effect of the number of boundaries on the mechanical properties. It is traditionally believed that grain boundaries are barriers to the propagation of dislocations and prevent elastoplastic deformation. However, it was also shown that boundaries can play an alternative role and be sources of dislocations. As an example of the dual role of the grain boundary, we provide the data of [43]. It should be noted that the second case (boundaries are sources of dislocations) is valid for well-annealed crystals with a low content of dislocations. It is more likely that the first case where boundaries are barriers is valid for the present work. In addition, a decrease in the number of dislocations helps to reduce internal stresses in the system. Thus, the mechanical properties should decrease in accordance with the grain size. However, this only occurs in volume, that is, other process act on the surface.

**Figure 4:**
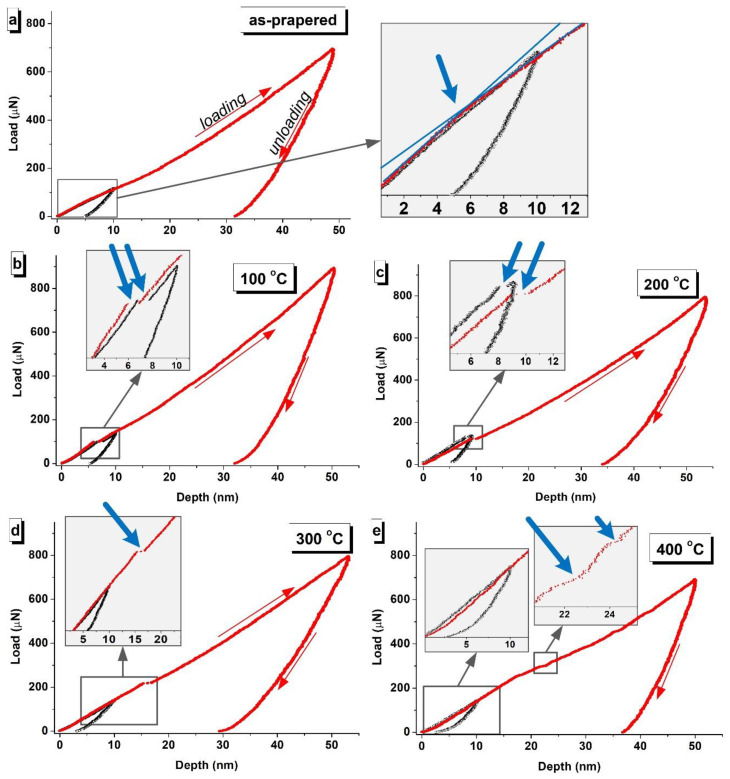
Characteristic deformation curves (loading-unloading) obtained by nanoindentation at the depth of 10 nm (black dots) and 50 nm (red dots) of the (**a**) as-prepared Au/NiFe nanostructured system and the same system after heat treatment with (**b**) 100 °C, (**c**) 200 °C, (**d**) 300 °C, and (**e**) 400 °C. The blue arrows indicate the moment the indenter passes through the interface of oxide and metal.

It is obvious that heat treatment in air activates surface oxidation. Typically, an oxide differs in the mechanical characteristics from a non-oxide metal. It is usually characterized by higher hardness and at the same time its embrittlement due to the large number of dislocations. Figure 4 shows typical indentation curves representing the dependence of the penetration depth on the indentation load. In a first approximation, the hardness depends on the tilt angle of the loading curve (the greater the tangent of the tilt angle, the higher the hardness). A detailed analysis of the deformation curves made it possible to determine the point on the loading part of the curve corresponding to the transition of the indenter from a harder to a softer layer. Accordingly, when testing the as-prepared Au/NiFe system at a depth of about 5 nm, the slope of the curve changes, as can be seen in Figure 4a, as well as in the enlarged fragment. This behavior was recorded for the curves that were obtained at a full indentation depth of 10 and 50 nm.

The gap appears at a depth of 5–7 nm at heat treatment temperature of 100 °C (Figure 4b). Similar behavior (gaps in the load curve) is also characteristic of cracks, but, in this study, a visually distinguishable change in the tilt angle of the curve before and after the gap is also observed. A similar phenomenon was described in the study [44]. Therefore, we can assume that the thickness of the oxide layer (*h_ox_*) after heat treatment at 100 °C is *h_ox_* = 5–7 nm. The same change in the gap and tilt exists on the loading curves of the sample after heat treatment at 200 °C, but with a shift to a greater depth, about 8–10 nm. This indicates an increase in the oxide thickness. The gap is at a depth of 14–18 nm for the curves that correspond to heat treatment at 300 °C. That is, when heat treatment at 300 degrees for 1 h, an oxide layer is formed with a thickness of *h_ox_* = 14–18 nm. There are no gaps in all of the analyzed curves (characteristic ones are presented here) after heat treatment at 400 °C. This probably indicates a diffuse interface between the oxidized and non-oxidized metal. 

According to the analysis of the indentation curves, it can be concluded that the hardness of the near-surface layer (*h* = 10 nm) correlates with the thickness of oxide h_ox_. When the thickness of oxide h_ox_ is less than the indentation depth (*h_ox_* < *h*), the hardness value includes two components: hardness of oxide and NiFe. This ratio holds for the as-prepared sample, after heat treatment at 100 and 200 °C. In this temperature range, the hardness increases from 8.6 to 11.0 GPa. When *h_ox_* > *h*, (after heat treatment at 300 and 400 °C), the hardness value remains constant at approximately 11.0 GPa, since the indentation takes place inside the oxide layer. Nevertheless, the obtained hardness value cannot be considered as oxide hardness, because the recommended indentation depth, which is necessary to exclude the influence of the substrate, is exceeded. The Young’s modulus continuously increases with increasing oxide thickness. The reason for the increase is that the oxide is a solid thin film on a soft sublayer (non-oxidized NiFe).

**Figure 5:**
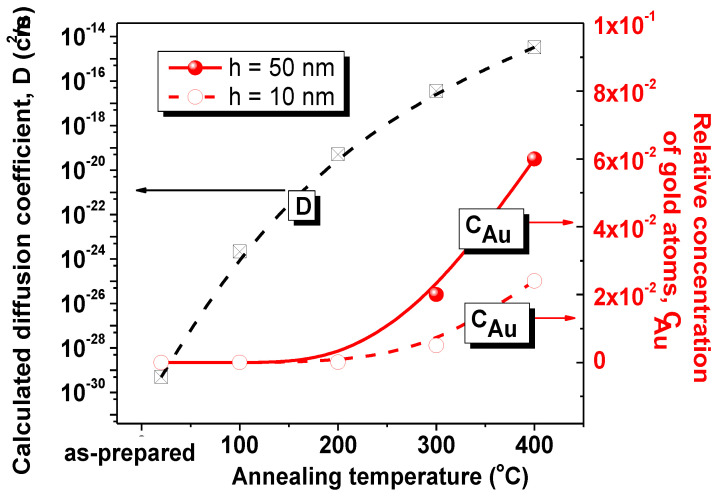
Effect of heat treatment temperature on gold atoms diffusion from a sublayer into the NiFe film: calculated values of the diffusion coefficient and relative concentration of gold atoms at a distance of 550 nm (or *h* = 50 nm) and 590 nm (or *h* = 10 nm) from the Au/NiFe interface.

It is well known that high temperature increases the mobility of atoms. The diffusion of atoms on both sides of the interface and self-diffusion are activated. Based on the Arrhenius equation, the thermal diffusion coefficient (*D*) is calculated, which characterizes the diffusion intensity of Au atoms from the sublayer into NiFe films. Figure 5 shows the results of calculations.

It was found that, at room temperature, *D* = 4.95 × 10^−30^ cm^2^/s. An increase in temperature to 100 °C causes an increase in diffusion coefficient to 2.18 × 10^−24^ cm^2^/s. Subsequently, *D* increases to 5.09 × 10^−20^, 3.56 × 10^−17^, 3.32 × 10^−15^ cm^2^/s for 200, 300, and 400 °C, respectively. I Fik’s law was used to calculate the relative concentration of gold atoms in the NiFe film (*C*) at a distance of 10 and 50 nm from the surface (or 590 and 550 nm from the Au/NiFe interface). It was found that the relative concentration of gold is negligible—less than 0.0001—at heat treatment temperature *T* ≤ 200 °C. The relative concentration becomes 0.02 at a depth of 50 nm (or 550 nm) and 0.005 at a depth of 10 nm (590) after heat treatment during 1 h at *T* = 300 °C. Thermal diffusion becomes even more intense at *T* = 400 °C and *C* = 0.06 at a distance of 50 nm and *C* = 0.02 at a distance of *h* = 10 nm.

Impurity atoms, which are defects in the crystal structure, as a rule, contribute to the propagation of dislocations during plastic deformation. A concentration of impurity atoms of approximately 2 or 6% (as in NiFe after 300 and 400 °C heat treatment) can significantly disorder the structure and lead to a decrease in the mechanical properties. Obviously, a high concentration of defects (or impurity atoms) in combination with an increase in the grain size leads to a decrease in the mechanical properties in the bulk of NiFe. In addition, impurity atoms can soften the surface, acting in opposite directions with hardening due to surface oxidation. This might limit the increase in hardness of the near-surface layer after heat treatment at *T* > 200 °C. 

## 4. Conclusions

A nanostructured NiFe film was obtained on single-crystal silicon with a gold sublayer by pulsed electrodeposition. An ultrashort pulse duration (10^−3^ s) led to the formation of a NiFe film with a grain size of 58 nm. The Au/NiFe system was heat treated in the temperature range from 100 to 400 °C in order to study the effect of heat treatment on the microstructure and mechanical properties of the two-dimensional system. Atomic force microscopy was used for the microstructure investigation. It was found that there are two stages of grain coalescence under the influence of heat treatment. At the same time, the grain size nonlinearly increases to 580 nm with an increase in temperature to 400 °C. The mechanical properties of the Au/NiFe system before and after heat treatment were studied while using high-resolution nanoindentation. The mechanical tests were carried out at two indentation depths—10 and 50 nm to study the influence of heat treatment not only on the film volume, but also on the near-surface layer. It was shown that heat treatment has an opposite effect on the hardness and Young’s modulus of the near-surface layer and the internal volume. The hardness and Young’s modulus of the as-prepared sample on the surface and in the volume are quite close, *H*_10nm_ = 8.64 ± 0.43 GPa, *H*_50nm_ = 7.51 ± 0.32 GPa, and *E*_10nm_ = 183.6 ± 7.0 GPa, *E*_50nm_ = 196.8 ± 5.4 GPa. For the near-surface layer, an increase in heat treatment temperature to 400 °C leads to a nonlinear increase in hardness to 11.1 ± 0.98 GPa and Young’s modulus to 256.1 ± 8.3 GPa (for 400 °C). For the internal volume of the NiFe film, there is a decrease in these parameters to *E*_50nm_ = 165.1 ± 9.3 GPa and *H*_50nm_ = 5.88 ± 0.58 GPa. 

A comprehensive study of processes that were activated by temperature and influenced the mechanical properties was carried out.

1. It has been established that an increase in grain size with a simultaneous decrease in the number of grain boundaries leads to a decrease in the number of barriers to the distribution of dislocations during mechanical deformation. Consequently, the mechanical properties decrease with increasing grain size, especially in the volume of the material, since the surface as a whole is characterized by an incomparably greater defectiveness, and other mechanisms act because of this.
2. Heat treatment in air activates surface oxidation. The oxidation process was studied step by step with increasing temperature while using an analysis of deformation curves. The thickness of the oxide layer increases from about 5 to 20 nm with an increasing temperature of heat treatment. It was found that when the oxide thickness h_ox_ is less than the indentation depth (*h_ox_* < *h*), the hardness value includes two components: hardness of oxide and NiFe. This is true for the range from the as-prepared sample to the sample after treatment at 200 °C. Hardness increases from 8.6 to 11.0 GPa in this range. When *h_ox_* > *h*, (after treatment at 300 and 400 °C), the hardness value remains constant at about 11.0 GPa, since the indentation takes place inside the oxide layer. It was also found that surface oxidation does not significantly affect the mechanical properties of the internal volume of the NiFe film.
3. The third process, which is activated by heat treatment, is the diffusion of Au atoms from the sublayer into the NiFe film. Diffusing atoms are point defects, which facilitate the propagation of deformation and soften NiFe. The gold concentration for each temperature at the investigated indentation depth (10 and 50 nm) was calculated using I Fick’s law and the Arrhenius equation. Consequently, it was found that a relative concentration of more than 0.02 is a critical point, after passing through which a decrease in hardness, elastic modulus, and resistance to elastoplastic deformation begins.

In addition, it was experimentally established that heat treatment of two-dimensional (2D) nanostructured Au/NiFe systems at 200 °C leads to an increase in the resistance to elastoplastic deformation in the near-surface layer without reducing the mechanical properties in the internal volume, due to moderate surface oxidation and a decrease in the internal stresses of nanoscale grains.

## Figures and Tables

**Table 1:** Technological parameter of the electrodeposition of the NiFe layer.

Parameter		Value
**Electrolyte composition, g/L**	**NiSO_4_**	210
	**NiCl_2_**	20
	**H_3_BO_3_**	30
	**MgSO_4_**	60
	**FeSO_4_**	15
	**Saccharin**	1
**Electrolyte pH**		2.3–2.5
**Electrolyte temperature, °C**		30–33
**Anodes**		Ni
**Current**		pulsed
**Pulse duration, s**		10^−3^
**Pause duration, s**		10^−3^
**Current density, mA/cm^2^**		25
**Deposition time, s**		300
**Effective deposition time, s**		150

**Table 2:** Features of the as-prepared Au/NiFe nanostructured system.

Parameter	Value
**Substrate**	Si wafer (100)
**Thickness of Au layer, nm**	100
**Thickness of NiFe layer, nm**	600
**Fe content, at.%**	24.45
**Ni content, at.%**	75.55
**Type of crystal structure (NiFe)**	cubic face-centered
**Space group**	Fm3m (No. 225)
**Unit cell parameter, Å**	3.573
